# Characterization of genomic alterations and neoantigens and analysis of immune infiltration identified therapeutic and prognostic biomarkers in adenocarcinoma at the gastroesophageal junction

**DOI:** 10.3389/fonc.2022.941868

**Published:** 2022-11-11

**Authors:** Yueqiong Lao, Yuqian Wang, Jie Yang, Tianyuan Liu, Yuling Ma, Yingying Luo, Yanxia Sun, Kai Li, Xuan Zhao, Xiangjie Niu, Yiyi Xi, Ce Zhong

**Affiliations:** Department of Etiology and Carcinogenesis, National Cancer Center/National Clinical Research Center/Cancer Hospital, Chinese Academy of Medical Sciences and Peking Union Medical College, Beijing, China

**Keywords:** adenocarcinoma at the gastroesophageal junction, genome and transcriptome, tumor neoantigens, CD8+ T cells, therapeutic biomarkers, prognostic prediction

## Abstract

**Objectives:**

Adenocarcinoma at the gastroesophageal junction (ACGEJ) refers to a malignant tumor that occurs at the esophagogastric junction. Despite some progress in targeted therapies for HER2, FGFR2, EGFR, MET, Claudin 18.2 and immune checkpoints in ACGEJ tumors, the 5-year survival rate of patients remains poor. Thus, it is urgent to explore genomic alterations and neoantigen characteristics of tumors and identify CD8+ T-cell infiltration-associated genes to find potential therapeutic targets and develop a risk model to predict ACGEJ patients’ overall survival (OS).

**Methods:**

Whole-exome sequencing (WES) was performed on 55 paired samples from Chinese ACGEJ patients. Somatic mutations and copy number variations were detected by Strelka2 and FACETS, respectively. SigProfiler and SciClone were employed to decipher the mutation signature and clonal structure of each sample, respectively. Neoantigens were predicted using the MuPeXI pipeline. RNA sequencing (RNA-seq) data of ACGEJ samples from our previous studies and The Cancer Genome Atlas (TCGA) were used to identify genes significantly associated with CD8+ T-cell infiltration by weighted gene coexpression network analysis (WGCNA). To construct a risk model, we conducted LASSO and univariate and multivariate Cox regression analyses.

**Results:**

Recurrent *MAP2K7*, *RNF43* and *RHOA* mutations were found in ACGEJ tumors. The COSMIC signature SBS17 was associated with ACGEJ progression. *CCNE1* and *VEGFA* were identified as putative CNV driver genes. *PI3KCA* and *TP53* mutations conferred selective advantages to cancer cells. The Chinese ACGEJ patient neoantigen landscape was revealed for the first time, and 58 potential neoantigens common to TSNAdb and IEDB were identified. Compared with Siewert type II samples, Siewert type III samples had significant enrichment of the SBS17 signature, a lower *TNFRSF14* copy number, a higher proportion of samples with complex clonal architecture and a higher neoantigen load. We identified 10 important CD8+ T-cell infiltration-related Hub genes (*CCL5*, *CD2*, *CST7*, *GVINP1*, *GZMK*, *IL2RB*, *IKZF3*, *PLA2G2D*, *P2RY10* and *ZAP70*) as potential therapeutic targets from the RNA-seq data. Seven CD8+ T-cell infiltration-related genes (*ADAM28*, *ASPH*, *CAMK2N1*, *F2R*, *STAP1*, *TP53INP2*, *ZC3H3*) were selected to construct a prognostic model. Patients classified as high risk based on this model had significantly worse OS than low-risk patients, which was replicated in the TCGA-ACGEJ cohort.

**Conclusions:**

This study provides new neoantigen-based immunotherapeutic targets for ACGEJ treatment and effective disease prognosis biomarkers.

## Introduction

There were over 1.6 million new cases of esophagogastric (esophageal, gastroesophageal junction or gastric) cancer and 1.3 million related deaths worldwide in 2020, with esophagogastric cancer ranking third in terms of incidence and second in terms of mortality (1). The incidence of adenocarcinoma of the gastroesophageal junction (ACGEJ) has increased rapidly worldwide in the past four decades (2). According to the widely used Siewert classification, ACGEJ is divided into three subtypes based on the location of the tumor epicenter (3). Siewert type I is treated as esophageal cancer, while Siewert types II and III are considered true gastric cardia tumors and gastric tumors. The National Comprehensive Cancer Network (NCCN) guidelines recommend surgery for patients with early-stage ACGEJ, preoperative chemoradiation for patients with locally advanced ACGEJ, and trastuzumab plus chemotherapy for patients with HER2-positive metastatic ACGEJ (4, 5). Immune checkpoint inhibitor (ICI) treatments, such as nivolumab and pembrolizumab (anti-PD-1 antibodies), have shown encouraging efficacy in patients with unresectable advanced or metastatic ACGEJ (6). However, the prognosis of patients with advanced-stage gastric cancer and ACGEJ remains poor, with a median overall survival (OS) of approximately 1 year (6). Multiple factors, including tumor-specific mutant antigens (neoantigens) and the tumor immune microenvironment (TME), may affect the effectiveness of ICIs. Recently, a study from China demonstrated that Siewert type I tumors and Siewert type II/III tumors had different driver genes, mutational signatures and disrupted pathways (7). In our previous study, we performed whole genome and transcriptome sequencing in 124 Chinese ACGEJ tumors and categorized them as tumor with chromosomal instability dominated by CNVs and identified alterations vulnerable to drugs and that could be used as prognostic biomarkers for ACGEJ patients (8). However, comprehensive molecular characteristics, especially those of tumor neoantigens, and of different Siewert types in ACGEJ remain unclear. Therefore, it is urgent to investigate the molecular characteristics of ACGEJ patients and identify tumor-specific targets for treatments and potential prognostic markers.

Neoantigens are generated by various mutation types, including nonsynonymous single nucleotide variants (SNVs), insertions or deletions (indels) and gene fusions (9, 10). Neoantigens can be loaded into MHC class I molecules and recognized by cytotoxic CD8+ T cells, which increase immune cell infiltration and enhance the efficacy of cancer immunotherapy (11, 12). Importantly, neoantigen load has been related to prolonged survival in patients with melanoma, non-small-cell lung cancer and mismatch repair-deficient colorectal cancer treated with ICIs (13–16). Neoantigens in ACGEJ can serve as excellent targets for immunotherapy. Many previous studies have revealed that neoantigen vaccine treatment is inhibited by the TME, which impedes the function of immune cells and even hinders the immune response (17–26). For instance, regulatory T (Treg) cells can suppress the induction of effective neoantigen-specific T-cell responses in tumors (17). Neoantigen-specific resident memory T (TRM) cells induced by therapeutic vaccination may provide long-term immune surveillance and prevent disease recurrence (18). In addition, neoantigen vaccines spontaneously upregulate the expression of inhibitory receptors (such as PD-1 and TIM3) in CD4+ T cells and CD8+ T cells, indicating that functional blockade of these receptors contributes to the generation of efficient neoantigen-specific T-cell responses (19–23). Although few studies have reported the association of cancer- associated fibroblasts (CAFs) with neoantigen-targeted therapy, CAFs were identified as potential mediators of the response to immune checkpoint inhibitor (ICI) treatments (24–26). The combination of neoantigen vaccines with other therapies, such as ICIs, chemotherapy, radiotherapy and immunosuppressive factor-targeted therapies, generates a stronger antitumor response. Moreover, preclinical data demonstrated that the combination of the neoantigen vaccine VB10.NEO and NKTR-214, a T-cell-proliferation inducer, can induce clonal expansion of natural killer (NK), CD4+ T and CD8+ T cells and induce strong and durable neoantigen-specific T-cell responses (27). CD8+ T cells are central effector cells in the tumor immune microenvironment and are primarily responsible for recognizing and killing tumor cells. Several studies have investigated the relationship between intratumoral CD8+ T cells and the prognosis of gastric cancer, two of which reported that a high abundance of CD8+ T cells was associated with better OS in patients with gastric cancer (28–30). Unfortunately, we know little about the role of CD8+ T cells in ACGEJ. Exploring CD8+ T-cell infiltration-related genes can provide targets for immunotherapy that can enhance the antitumor effect of tumor neoantigen vaccines and biomarkers to predict the OS of patients in ACGEJ.

In this study, we explored the molecular characteristics of ACGEJ and compared SNVs, copy number variations (CNVs), clonal patterns, and neoantigen load between Siewert types II and III and among differentiation grades. We also searched for CD8+ T-cell infiltration-related Hub genes and conducted weighted gene coexpression network analysis (WGCNA) to identify prognostic markers for the construction of a risk prediction model.

## Materials and methods

### Biospecimen collection

This study included 55 Chinese ACGEJ patients seen at the Linzhou Cancer Hospital and Linzhou Esophageal Cancer Hospital (Henan Province, China) from 2014 to 2016. All patients received no treatments before surgery and signed written informed consent forms. ACGEJ tumor, tumor-adjacent (> 5 cm from the tumor margins) and normal tissues and peripheral blood samples were collected during surgery. Pathological diagnoses were independently confirmed by at least two pathologists. Clinical information of patients was collected from medical records. Clinical follow-up data were obtained by phone interview, the most recent interview was conducted in September 2021 and the median follow-up period of patients was 50.3 months. This research was approved by the Institutional Review Board of Cancer Hospital, Chinese Academy of Medical Sciences, and Peking Union Medical College.

### Whole-exome sequencing

Genomic DNA was extracted from 55 matched tumor and blood samples using the Allprep DNA Kit (Qiagen) and QiaAmp Blood Midi Kit (Qiagen). Exon target capture was performed using an Agilent SureSelect Human All ExonV5 kit (Agilent) following the manufacturer’s protocol. After quality checking, the libraries were sequenced on an Illumina HiSeq xTen with 2X 150 bp paired-end reads. Average sequencing depths of 380x and 130x were achieved for paired tumor and blood samples, respectively (**Supplementary Tables 1**, **2**). Importantly, WES data in 55 Chinese ACEGJ patients have not been published.

### Data collection from database

We obtained somatic mutation data of 105 The Cancer Genome Atlas (TCGA) ACGEJ samples from the Broad Institute GDAC Firehose website (https://gdac.broadinstitute.org/) (31) and an additional 40 ACGEJ samples from the Tumor Portal (http://www.tumorportal.org) (32) (**Supplementary Table 3**). Transcriptomic data (FASTQ format) of the 55 Chinese ACGEJ patients described above were derived from our earlier publication (33). Combining two published studies of our group on ACGEJ tumors (8, 33), we obtained bulk RNA sequencing data of 178 ACGEJ samples, which consisted of 123 patients from one cohort (8) and 55 patients from the other (33). We conducted follow-up telephone interviews until September 2021, with 47.7% (85/178) of patients being lost. Eventually, the gene expression (transcripts per million, TPM) data of 93 Chinese ACGEJ samples in a period of 50.3 months (median) were included and also publicly available from NCBI Gene Expression Omnibus (https://www.ncbi.nlm.nih.gov/geo, GSE159721). The gene expression (TPM) data of 86 TCGA ACGEJ samples (tumors located in the cardia/GEJ rather than other sites of the stomach (body of stomach, fundus of stomach, gastric antrum, pylorus, etc) and the corresponding clinicopathological information were obtained from the TCGA Pan-cancer Atlas (https://gdc.cancer.gov/about-data/publications/pancanatlas) (34).Moreover,we obtained the tumor-specific neoantigen data of 441 TCGA stomach adenocarcinoma (STAD) samples from TSNAdb (http://biopharm.zju.edu.cn/tsnadb) (35). We then downloaded a list of gastric adenocarcinoma neoepitope that bound to MHC class I molecules from the Immune Epitope Database (IEDB) (36).

### Somatic mutation identification

DNA sequence reads were mapped to the human reference genome GRCh37 (Ensembl) using BWA-MEM (v0.1.22) (37) with default parameters. Somatic SNVs and indels in each tumor sample were called by Strelka2 (v2.8.3) (38) and annotated by Ensembl Variant Effect Predictor (VEP, release 90) (39). Low-quality and potential germline variants were filtered by gatk-tools (v0.2.2).

### Identification of mutational signatures

Based on nonnegative matrix factorization (NMF), SigProfiler was applied to identify single-base-substitution (SBS) mutational signatures (40). Six base substitutions (including C>A, C>T, C>G, T>A, T>C, T>G) and their trinucleotide sequence context were considered in this analysis. We used SigProfilerMatrixGenerator and SigProfilerExtractor to categorize the SNV mutations and then extract *de novo* mutational signatures. After matching to COSMIC signatures, we obtained 5 mutational signatures and determined the contribution of each signature in each individual sample.

### Copy number alteration analysis

FACETS (v0.5.14) (41), an allele-specific copy number analysis tool, was used to estimate integer copy number calls corrected for tumor purity, ploidy and clonal heterogeneity. VCF files from FACETS were used as input for GISTIC2.0 (v.2.0.23) (42) to identify significantly amplified or deleted regions of the genome for each patient. The Seg.CN value required by GISTIC2.0 was calculated with TCN_EM values from FACETS as Seg.CN= log2(TCN_EM) -1. Arm- and focal-level CNV regions with FDR q< 0.25 were considered significantly aberrant regions.

### Clonality analysis of somatic mutations

We used the R package SciClone (43) to infer clonal and subclonal architectures by clustering similar variant allele frequencies of somatic mutations in a single sample using a variational Bayesian binomial mixture model. The regions of CNVs and loss of heterozygosity (LOH) inferred by FACETS and somatic mutations of sufficient depth (20x or greater coverage) called by Strelka2 from WES were used as inputs. Due to lack of enough points to cluster or lack of copy number neutral regions, two ACGEJ specimens (GEJ183 and GEJ191) were excluded; subsequently, a total of 53 samples were analysed.

### Neoantigen prediction

We used WES data of blood samples or tumor-adjacent normal tissues in FASTQ format to perform human leukocyte antigen (HLA) typing. OptiType (v1.3.5) (44) was applied to predict class-I HLA typing with default settings. The raw RNA sequencing (RNA-seq) data of tumor tissues in FASTQ format were processed by Kallisto (v0.46.0) (45) to obtain the expression values (TPM) with the GRCh38 v78 coordinates. Tumor-specific somatic variant calls, RNA-seq expression values and class-I HLA genotyping were given as inputs to the MuPeXI pipeline (v1.2) (46) to predict 9 amino acid peptides. Notably, somatic mutation calls from the GRCh37 alignment were converted to GRCh38 by running liftover in MuPeXI. The binding affinities of a mutant peptide with the major histocompatibility complex class I (MHC-I) molecules of patients were predicted by the NetMHCpan (v4.0) (47) algorithm in MuPeXI. Mutant peptides with an eluted ligand percentile rank (EL% rank) score ≤ 2% and RNA expression level (TPM) > 0.1 were predicted to be neoantigens. Neoantigens with a percentile rank score< 0.5% were considered high-affinity neoantigens.

### Immune cell infiltration estimation

MCP-counter (48), which uses a deconvolution approach, was applied to produce the absolute abundance scores for endothelial cells and fibroblasts as well as 8 immune cell types, including CD8+ T cells. xCell, another cell type enrichment analysis method, was used to quantify the relative abundance scores for immune and stromal cells (49).The Tumor Immune Estimation Resource 2.0 (50) (TIMER2.0, http://timer.cistrome.org/) web server is a comprehensive resource integrating multiple immune infiltration estimation algorithms, including MCP-counter and xCell. In this study, we imported the TPM-normalized gene expression matrix of ACGEJ samples into TIMER2.0 and obtained the MCP-counter-based and xCell-based deconvolution profiles of CD8+ T cells.

### Construction of the coexpression network and identification of significant modules

The WGCNA (51) package was used to construct a weighted gene coexpression network of the 6,000 most variable genes based on RNA-seq data (based on median absolute deviation) among ACGEJ samples. A total of 93 tumor samples from our previously published data were included after removing one outlier specimen with sample hierarchical clustering. The gene expression matrix was converted into a similarity matrix, and the appropriate weighting coefficient β was chosen. Subsequently, an adjacency matrix was generated and transformed into a topological overlap matrix (TOM). Next, we performed hierarchical clustering with the dynamic tree cut method and identified modules with the following parameters: minModuleSize = 30 and mergeCutHeight = 0.25. Afterwards, we calculated the correlation between module eigengenes (MEs) and clinical traits to identify clinically significant related modules (P< 0.05). In general, the turquoise module was considered the key module.

### Functional enrichment analysis

To investigate the biological functions of turquoise module genes, we conducted gene ontology (GO) and Kyoto Encyclopedia of Genes and Genomes (KEGG) pathway enrichment analyses by using the clusterProfiler R package (v3.14.3) (52). The thresholds were set as *P<* 0.01 and FDR q< 0.05.

### Hub gene identification

Hub genes, highly interconnected with the nodes in a given module, are generally determined based on gene significance (GS) and module membership (MM). GS is defined as the correlation between the gene and a clinical trait, while MM is described as the correlation between the gene and a module. In this study, genes with GS > 0.6 and MM > 0.8 were selected as Hub genes. We further used the CytoHubba and MCODE functions of Cytoscape software (v 3.8.2, https://cytoscape.org/) to select and visualize the 10 most important Hub genes in the turquoise module.

### Construction and validation of a risk prediction model

A total of 179 patients with ACGEJ from two independent cohorts were included in our study. Bulk RNA-seq data and clinical information were collected. Based on the turquoise module genes, we developed a risk prediction model in the training cohort (containing 93 ACGEJ samples from our previous studies) and validated it in an external cohort (containing 86 ACGEJ samples from the TCGA database). In the training set, we conducted univariate Cox regression analysis to identify the genes that were closely associated with the OS of patients (*P*<0.05). Subsequently, least absolute shrinkage and selection operator (LASSO) Cox regression analysis was performed to narrow down the list of candidate genes. Finally, multivariate Cox proportional hazards regression analysis was used to select independent prognostic genes and construct the risk score model. The risk score was calculated using the following formula:


$$\text{risk score}=\sum \left(\text{expression}{\text{ mRNA}}_{i}\times {\text{coefficient}}_{i}\right)$$

According to the median value of the risk score, we stratified patients into high-risk and low-risk groups and performed Kaplan–Meier analysis (with log-rank test) to compare the survival differences. Additionally, the performance of the model was evaluated by area under the curve (AUC) analysis using the “ROCR” package in R.

### Statistical analyses

All statistical analyses were performed using R3.6.3. The Wilcoxon rank-sum test was utilized to compare nonnormally distributed continuous variables between two groups. Categorical data were compared using Fisher’s exact test. Spearman’s rank correlation analysis was applied to measure the correlation between two continuous variables. Based on the correlation between gene expression and patient survival, the “surv-cutpoint” function in the survminer R package was used to find the optimal cut-off point. The survival curves for the prognostic analysis were created by the Kaplan–Meier method, and log-rank tests were conducted to evaluate the significance of differences. Statistical significance was described as follows: ns, not significant; *P< 0.05; **P< 0.01; **** *P*<0.0001.

## Results

### Driver genes of ACGEJ

We performed WES of tumor and matched blood samples from 55 Chinese ACGEJ patients (**Supplementary Table 4**) with a median age of 64 years (range 42−80 years old); the cohort included 41 males (74.5%). The majority of the patients had TNM stage III disease (67.3%). Moreover, 23.6% of patients had poorly differentiated disease, and 45.5% had Siewert III subtype disease. All tumors were microsatellite stable (96.4%) or had microsatellite instability (3.6%). To better understand the genomic alterations in ACGEJ, we downloaded and analysed the coding-region mutation data for 145 patients with ACGEJ from TCGA and the Tumor Portal database. The two cohorts were similar to our cohort in age, sex and microsatellite status though differed in tumor stage. There was a significantly higher proportion of stage II and IV patients in the TCGA/Tumor Portal cohort than in our ACGEJ cohort (45.5% *vs*. 21.8%, 10.3% *vs*. 0%, respectively; *P*< 0.05) and a lower proportion of stage III patients in the TCGA/Tumor Portal cohort (24.1% *vs*. 67.3%, *P<* 0.05).

**Figure 1:**
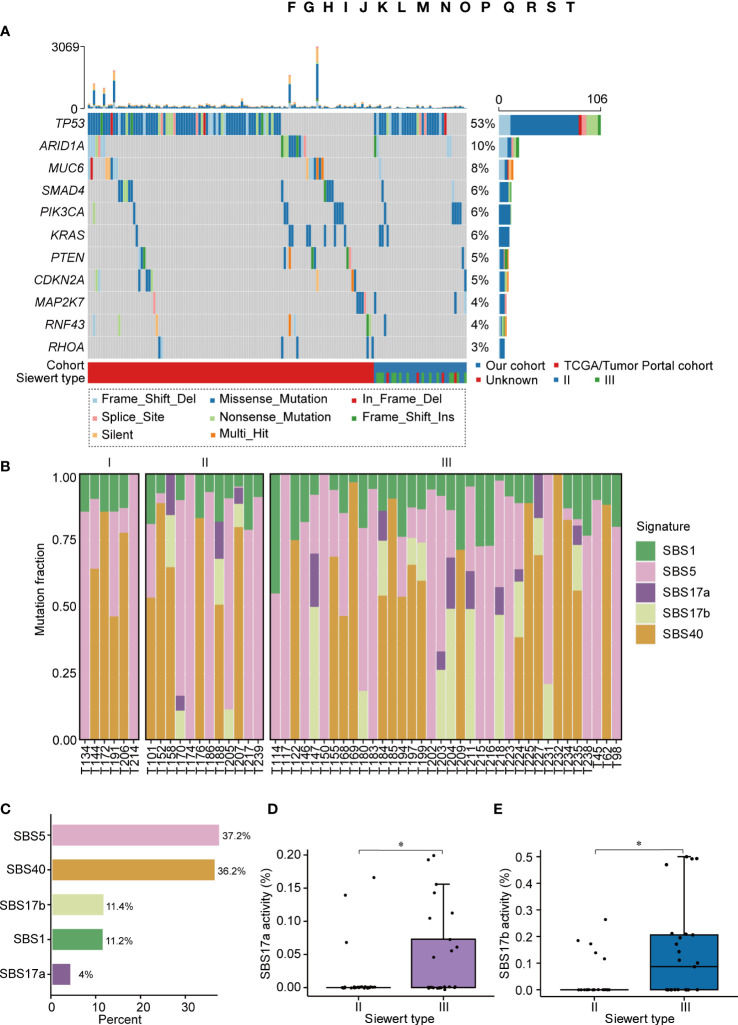
The landscape of somatic mutations and mutational signatures in ACGEJ. **(A)** The mutational landscape of driver genes in the 200 combined ACGEJ samples. Each column represents the tumor genome of one patient, and each row represents a gene. The top histogram shows the number of SNVs and indels in each patient. The histogram on the right displays the number of SNVs and indels in each gene. Driver genes (dNdScv, FDR *q*< 0.1) are ordered by the fraction of mutated tumor samples. **(B)** Bar plots showing the defined mutational signatures in our ACGEJ samples. Samples are ordered by TNM stage. **(C)** Bar plots comparing the defined mutational signatures and their relative contributions to somatic mutations. **(D, E)** SBS17a activity **(D)** and SBS17b activity **(E)** in different ACGEJ Siewert types. *P* values were derived from Wilcoxon rank-sum tests. Statistical significance was described as follows: *P < 0.05.

We then identified 4,018 somatic mutations (3,761 SNVs and 257 indels) in our ACGEJ specimens. The median tumor mutation burden (TMB) of our and TCGA/Tumor Portal cohorts were 1.04 (range 0.02−3.32) and 2.61 (range 0−67.94) per Mb, respectively, and the difference was significant (Wilcoxon rank-sum test, *P*< 0.0001; **Supplementary Figure 1A**). The TMB of Siewert type II cases was similar to that of Siewert type III cases, but there was no significant difference in TMB among the three differentiation grades (**Supplementary Figures 1B, C**). By combining two methods (dNdScv and MutSigCV), *TP53* (mutated in 52.7% of samples) was identified as the only significant driver gene in our ACGEJ cohort (FDR q< 0.1). To increase the detection power and accuracy, we combined our samples and the TCGA/Tumor Portal cohort samples (a total of 200 ACGEJ samples) and found 11 significant driver genes (FDR *q*< 0.1): *TP53*, *ARID1A*, *MUC6*, *SMAD4*, *PIK3CA*, *KRAS*, *PTEN*, *CDKN2A*, *MAP2K7*, *RNF43* and *RHOA*. (**Figure 1A**). Recurrent hotspot mutation analysis of our data and TCGA/Tumor Portal cohort data indicated that *TP53* had 3 common mutations: p.Arg248Gln, p.Arg248Trp and p.Arg273His (**Supplementary Figure 1D**). In TCGA/Tumor Portal tumors, the most frequent mutation of *TP53* was p.Arg175His, accounting for 13.2% (7/53) of the missense mutations. These results suggested that low driver mutation abundance is a dominant feature of Chinese ACGEJ samples due to their lower TMB.

### Mutational signature of ACGEJ

The predominant somatic mutation type was 5′-C [T > G] T-3′ in 96 substitution mutations in tumor samples from our cohort (**Supplementary Figure 1E**). To further explore the underlying mutational processes in ACGEJ tumors, we performed *de novo* single base substitution (SBS) mutational signature extraction and identified five mutational signatures, namely, SBS1, SBS5, SBS17a, SBS17b and SBS40 (**Figure 1B**). SBS1 and SBS5, which are common in tumors, are two clock-like and ageing-related endogenous mutational signatures, and they accounted for 48.4% of somatic mutations in ACGEJ (**Figure 1C**). SBS40, also correlated with ageing, accounted for 36.2% of somatic mutations in ACGEJ samples (**Figure 1C**). SBS17 (accounting for 15.4% of somatic mutations) can be split into SBS17a, defined by T > C substitution in the CTT trinucleotide context, and SBS17b, characterized by T > G conversion in any of the NTT trinucleotide contexts. Interestingly, we found that SBS17a and SBS17b were stage-related in ACGEJ patients, mainly appearing in TNM stage II and III patients, but were rare in stage I patients (**Figure 1B**). Compared with Siewert type II cases, Siewert type III cases showed significantly increased SBS17a and SBS17b signatures (both *P<* 0.05; **Figures 1D, E**). These results indicate that SBS17 may be related with the progression of ACGEJ.

### Somatic CNVs and functionally aberrant pathways in ACGEJ

**Figure 2:**
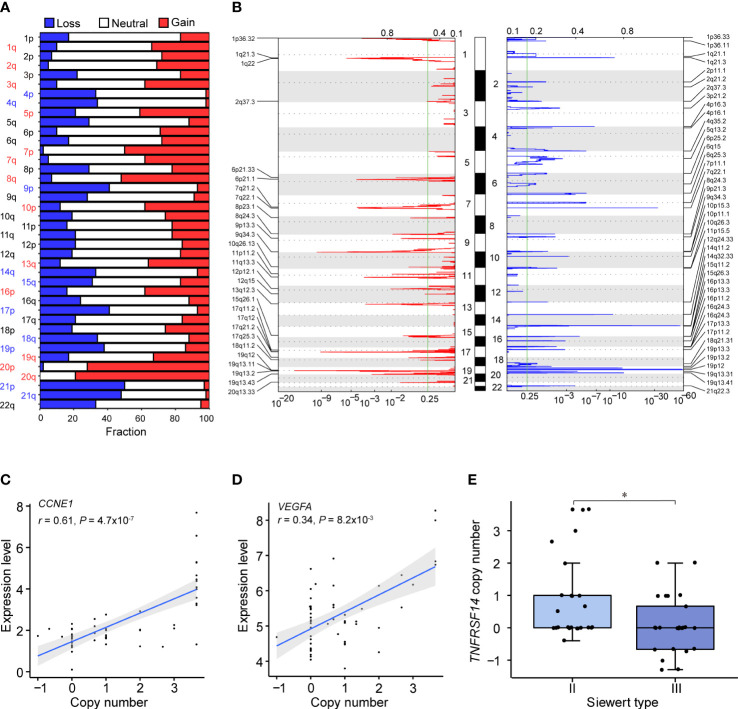
Somatic CNVs identified in our ACGEJ samples. **(A)** CNVs at the chromosome arm level. The bar graphs show the frequency of arm-level copy-number alterations, and the horizontal axis represents chromosome arms. Significant chromosome arm gains and losses (*q*< 0.25 and frequency > 30%) are indicated in red and blue, respectively. **(B)** Focal peaks of amplifications (left) and deletions (right) detected by GISTIC 2.0. The x axis represents the false discovery rate, and the y axis represents the chromosome. The green line represents the significance threshold (*q* = 0.25). **(C, D)** Correlations between the copy number and expression level of two putative CNV driver genes, *CCNE1* (left) and *VEGFA* (right). The copy number data on the horizontal axis were derived from the GISTIC output (all_data_by_genes.txt file). *P* values were derived from Spearman’s correlation tests. **(E)** Comparison of *TNFRSF14* copy number between Siewert II and III samples. *P* values were derived from Wilcoxon rank-sum tests. Statistical significance was described as follows: *P < 0.05.

In our ACGEJ tumors, we identified several chromosome arm level CNVs, including gains of 1q, 2q, 3q, 5p, 7p, 7q, 8q, 10p, 13q, 16p, 19q, 20p and 20q and losses of 4p, 4q, 9p, 14q, 15q, 17p, 18q, 19p, 21p and 21q (all q< 0.25 and frequency > 30%; **Figure 2A** and **Supplementary Table 5**). We also identified 29 recurrent amplification peaks and 43 recurrent deletion peaks (q< 0.25), for which 1,153 and 1,300 genes overlapped, respectively (**Figure 2B** and **Supplementary Tables 6**, **7**). Among specimens with different Siewert types or differentiation grades (**Supplementary Figures 2A, B**), we found similar gene-level CNVs. Among the 2,453 genes located in these CNV regions, 268 (10.9%) with copy number gain and 233 (9.5%) with copy number loss had mRNA levels significantly correlated with copy number (Spearman’s *r* > 0.3 and FDR q< 0.05). Intersection of the above-described genes and the driver gene *TP53* with the cancer-related genes in COSMIC yielded a list of 29 genes, which were found to be functionally involved in the p53/cell cycle and PI3K/AKT signalling pathways by KEGG analysis. As shown in **Supplementary Table 8**, p53/cell cycle pathway aberration was related to *TP53* mutation (52.7%) and amplifications of *CCNE1* (63.6%), *CDK6* (49.1%), *CASP8* (25.5%) and *MDM2* (21.8%) as well as deletion of *SMAD4* (40%). PI3K/AKT pathway aberration was related to amplifications of *FGFR2* (36.4%), *KRAS* (27.3%) and *CREB1* (23.6%). These results further confirmed the suggestion of our previous studies that *CCNE1* and *VEGFA*, which showed recurrent gene-level CNV, are CNV driver genes (FDR q ≤ 0.1, in ≥10% samples, **Figures 2C, D**). In addition to *ERBB2* amplification, a well-recognized target, other CNVs, such as *CCNE1* amplification and *VEGFA* amplification, could be targeted therapeutically. Moreover, we found that the copy number of *TNFRSF14* was significantly lower in Siewert type III than in Siewert II cases (**Figure 2E**, *P<* 0.05).

### Clonal and subclonal architectures of ACGEJ

**Figure 3:**
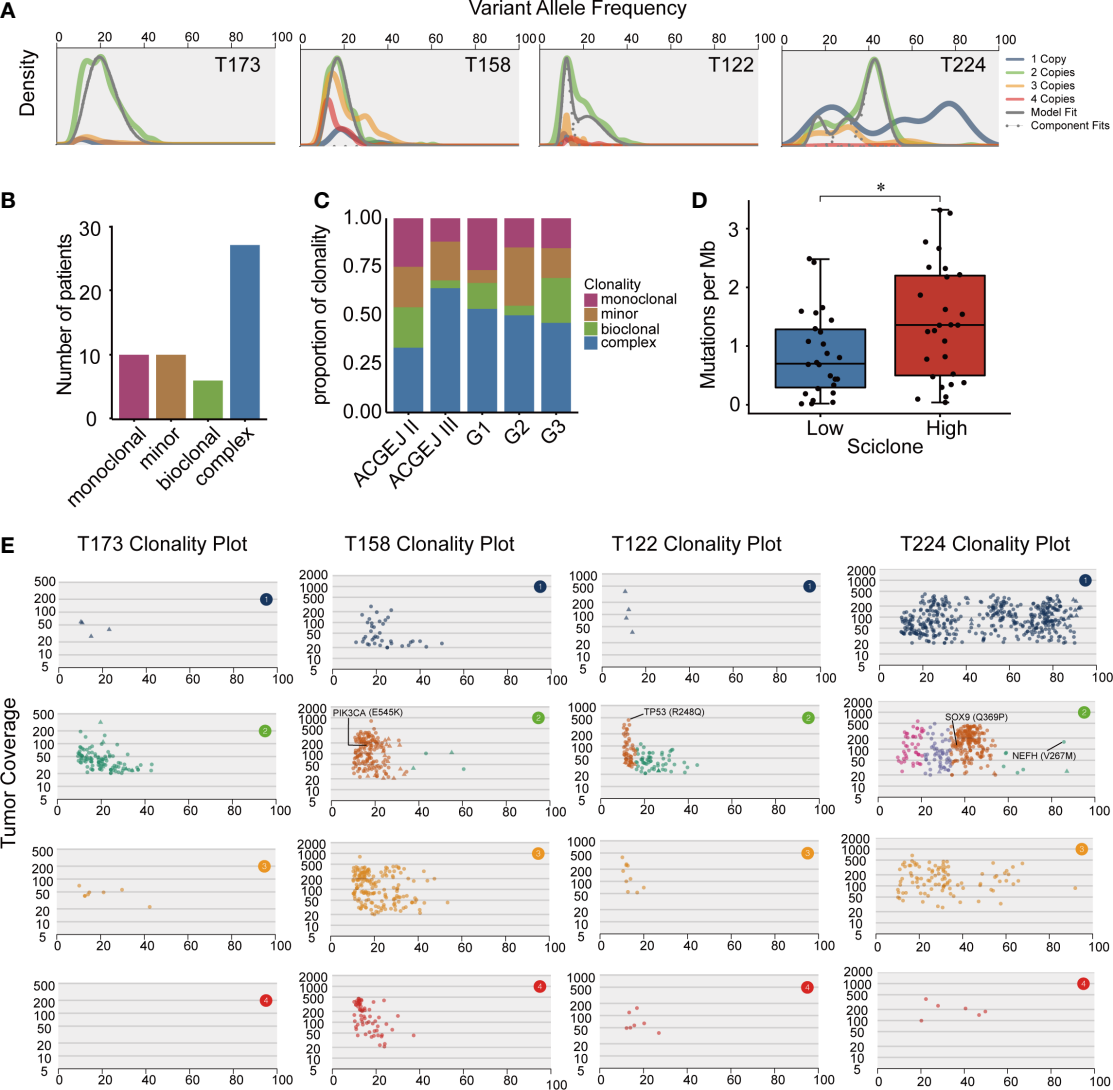
The clonal and subclonal architectures inferred in our ACGEJ samples. **(A)** Kernel density plots of variant allele frequency (VAF) across regions with one, two, three or four copies, posterior predictive densities summed over all clusters for copy-number-neutral variants and posterior predictive densities for each cluster/component. **(B)** Clonal patterns of 53 ACGEJ samples. **(C)** The distribution of clonal patterns across histopathological subtypes. ACGEJ II: Siewert type II, ACGEJ III: Siewert type III, G1: well differentiated, G2: moderately differentiated, G3: poorly differentiated or undifferentiated. **(D)** Comparison of TMB between patients with low and high intratumor heterogeneity defined by clonal patterns. **(E)** VAFs versus tumor coverage for each of the four copy number regions for four representative samples: T173, T158, T122 and T224. Mutations in previously reported driver genes are labelled. Statistical significance was described as follows: *P < 0.05.

Clonal and subclonal architectures reflect intratumor heterogeneity that may drive cancer progression and drug resistance. We used SciClone to reconstruct the clonal and subclonal architectures in each patient and obtained the number and genetic composition of clones. We identified four clonal patterns in 55 ACGEJ patients (**Figure 3A**), including monoclonal (a single dominant clone), minor subclone (a single dominant clone with a minor subclone), biclonal (two major clones) and complex (more than two clones) clonal patterns. The complex clonal pattern was the most common pattern in our samples (**Figure 3B**). We then analysed the distribution of the clonal patterns across samples with different histopathological features and observed that the complex clonal status was significantly higher in Siewert type III than in Siewert type II samples (64.0% *vs*. 33.3%, *P*< 0.05; **Figure 3C**). However, ACGEJ samples with good, moderate or poor differentiation grades exhibited similar clonal architectures. We then defined tumors with monoclonal, minor subclone and biclonal patterns as having low intratumor heterogeneity and those with complex clonal patterns as having high intratumor heterogeneity. As shown in **Figure 3D**, higher TMB was significantly associated with higher intratumor heterogeneity (*P*< 0.05). We further explored the clonality of recurrently mutated genes identified in ACGEJ or gastric cancer samples (7, 53–56) and found that mutations of *PIK3CA* (E545K), *TP53* (R248Q), *SOX9* (Q369P) and *NEFH* (V267M) were located in the dominant founding clone, indicating that mutations in these genes were acquired at an earlier time point (**Figure 3E**). These results emphasized the importance of *PI3KCA* and *TP53* mutations in the initiation of ACGEJ.

### Characteristics of neoantigens in ACGEJ samples

**Figure 4:**
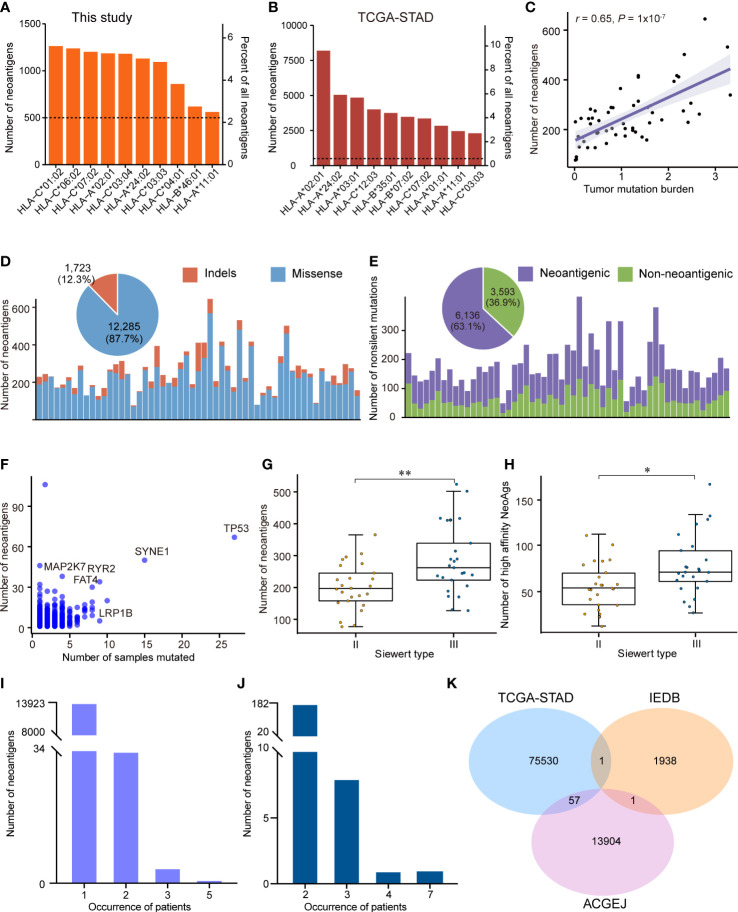
The neoantigen landscape in ACGEJ. **(A, B)** Ten predicted neoantigen sites of our ACGEJ samples **(A)** and TCGA-STAD samples **(B)**. **(C)** Correlations between neoantigen load and tumor mutation burden (Spearman’s correlation test). **(D)** Bar plots showing the number of neoantigens for each individual and their variant types. The pie plot on the top shows the fraction of variant types that produced neoantigens in 55 ACGEJ samples. **(E)** Bar plot and pie plot displaying the composition of nonsilent mutations that produced neoantigens. **(F)** Frequently altered genes with a high ability to generate neoantigens. The graph displays the number of samples containing specific genetic mutations and the relevant number of neoantigens. **(G, H)** Number of neoantigens **(G)** and number of high-affinity neoantigens **(H)** in different ACGEJ Siewert types. *P* values were derived from Wilcoxon rank-sum tests. NeoAgs: neoantigens. **(I, J)** Neoantigen recurrence in our patients **(I)** and TCGA-STAD patients **(J)**. **(G)** Venn diagram displaying the intersections of neoantigens from our ACGEJ patients, TCGA-STAD patients and IEDB. Statistical significance was described as follows: *P < 0.05; **P < 0.01.

Although genomic and transcriptomic alterations in ACGEJ have been characterized in some studies, the characteristics of neoantigens remain unclear. To the best of our knowledge, here, we describe the landscape of neoantigens in Chinese ACGEJ samples for the first time. We combined WES and RNA-seq data from our ACGEJ samples to predict neoantigens. First, we employed OptiType to identify the 4-digit HLA class I alleles in 55 ACGEJ samples and found that they were quite different from those in stomach adenocarcinoma (STAD) samples. HLA-C*01:02, HLA-C*06:02 and HLA-C*07:02 were the three most common neoantigen-binding sites in ACGEJ samples, while HLA-A*02:01, HLA-C*24:02 and HLA-C*03:01 were the top 3 in STAD samples (**Figures 4A, B**). Moreover, we identified 12,285 neoantigens originating from missense mutations and 1,723 neoantigens originating from frame-shift and in-frame indels, and 27.2% (3,808/14,008) of neoantigens were high-affinity neoantigens (binding affinity< 0.5% rank) (**Supplementary Table 9**). The neoantigen load in cancers usually shows a positive correlation with TMB (14, 16, 57). In ACGEJ, we found that the number of neoantigens was also positively correlated with TMB (*r* = 0.65, *P* = 1x10-7; **Figure 4C**). In STAD samples from TCGA, 2.09 neoantigens were generated per missense mutation and 2.53 neoantigens were generated per indel (10, 58). Similarly, we found that missense mutations generated fewer neoantigens (mean, 2.11 per mutation) than frame-shift indels (mean, 6.44 per mutation) in our specimens. In the ACGEJ samples, tumor neoantigens were mainly derived from missense mutations (**Figure 4D**), which accounted for 87.7% of neoantigens. Unsurprisingly, only 63.1% (45.6% - 76.4%) of nonsilent mutations generated neoantigens (**Figure 4E**), which indicated that amino acid sequence changes might not necessarily generate neoantigens. We further investigated whether frequently altered genes have the advantage of generating neoantigens and found that *TP53*, *SYNE1*, *MAP2K7*, *RYR2*, *FAT4* and *LRP1B* generate more neoantigens than other genes across all samples (**Figure 4F**). We also found that Siewert type III tumors possessed more neoantigens or high-affinity neoantigens than Siewert type II tumors (**Figures 4G, H**; *P*<0.05). However, there were no significant differences in neoantigen load or the number of high-affinity neoantigens among tumors of different TNM stages (I, II and III) (**Supplementary Figures 3A, B**). Similar to the results across TNM stage, neoantigen load and the number of high-affinity neoantigens were not significantly different among well, moderate and poorly differentiated tumors (**Supplementary Figures 3C, D**).

As the vast majority of the potential neoantigens that have been reported are patient-specific (58, 59), we next explored the heterogeneity of predicted neoantigens in ACGEJ. The results suggested that there were 13,962 unique neoantigens in 55 ACGEJ patients; 99.72% (13923/13962) of the neoantigens were found in only one patient, while only 0.28% (39/13962) of the neoantigens were found in at least two patients (**Figure 4I**). The most common share neoantigen was “GRQKRSDSL”, which was generated by *VSX1* (V138L). We also calculated the frequencies of neoantigens in TCGA-STAD samples from TSNAdb. Similarly, we found that there were 85,283 neoantigens in a total of 411 samples; only 0.21% (182/85,283) of the neoantigens were found in two samples, 8 neoantigens were found in three samples, one neoantigen was found in four samples, and one neoantigen was found in seven samples (**Figure 4J**). Furthermore, we compared the predicted neoantigens from our ACGEJ samples and TCGA-STAD samples to those found using IEDB data to identify common neoantigens (**Figure 4K** and **Supplementary Table 10**). We found 57 common neoantigens between the TCGA-STAD and ACGEJ samples, and only one neoantigen was common between the IEDB and ACGEJ samples. These results collectively point to the difficulty in ubiquitous neoantigen identification for ACGEJ.

### Construction of the weighted gene coexpression network and identification of Hub genes in ACGEJ

**Figure 5:**
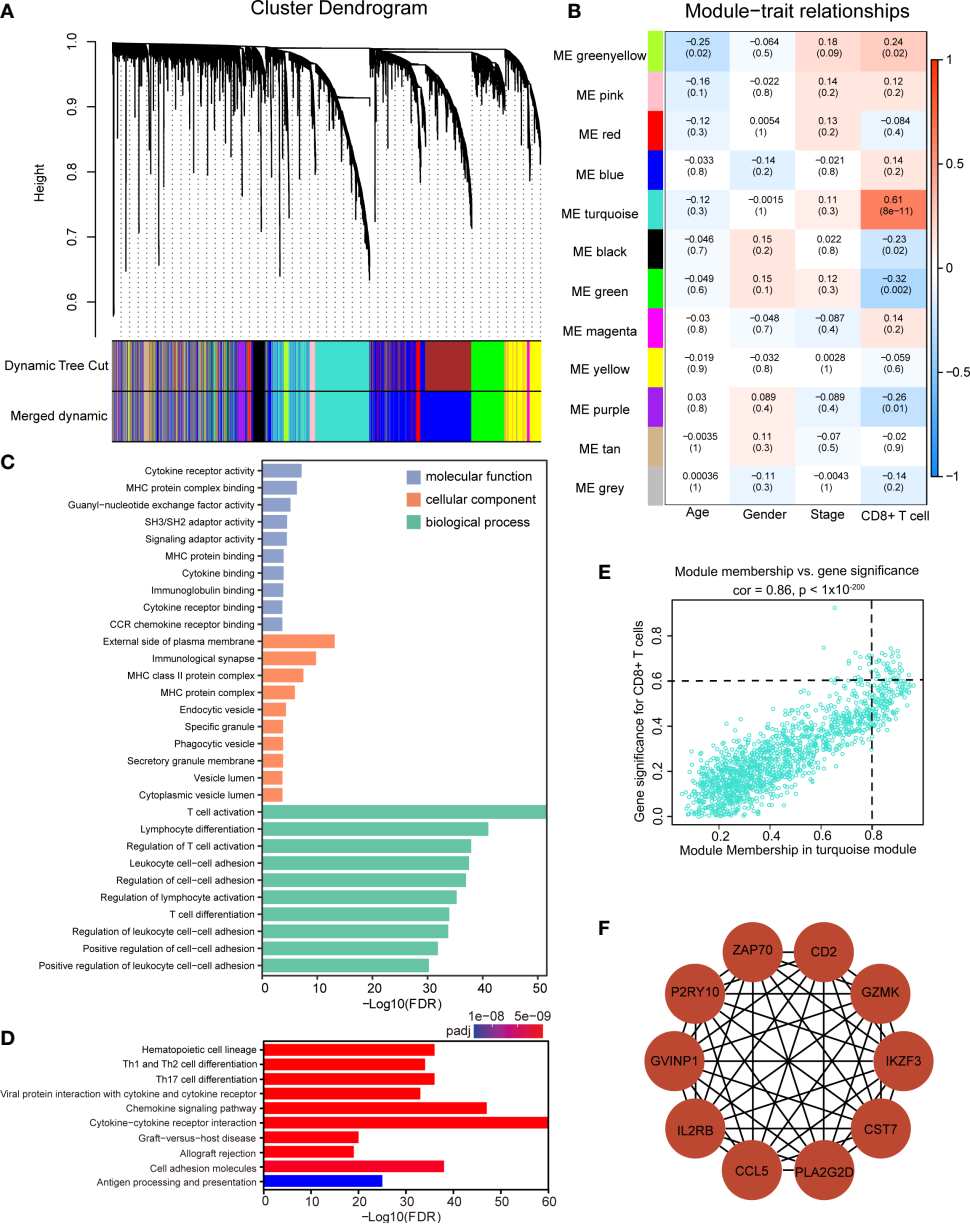
Identification of significant modules and Hub genes *via* WGCNA. **(A)** Gene dendrogram obtained by average linkage hierarchical clustering. The color row underneath the dendrogram indicates 12 different coexpression gene modules (the grey module represents unassigned genes. **(B)** The module–trait relationship heatmap between module eigengenes (row) and clinical traits (column). The correlation coefficients and p values are annotated in each box. **(C, D)** GO analysis **(C)** and KEGG pathway enrichment analysis **(D)** of all genes in the turquoise module. **(E)** Scatter plot of gene significance for the infiltration of CD8+ T cells (y axis) versus module membership (x axis) in the turquoise module. **(F)** Protein–protein interaction network of the ten Hub genes.

We examined all components in the TME, including immune and non-immune cells. Specifically, we applied xCell to estimate the abundance scores of immune and stromal cell populations in ACGEJ samples, and the results showed that the ratio of neoantigen to mutation was positively correlated with immune score, stromal score and microenvironment score (**Supplementary Figures 4A–C**). The ratio of neoantigen to mutation was significantly associated with TME cells, including B cell, myeloid dendritic cell, endothelial cell, cancer-associated fibroblasts, and T cell (**Supplementary Figures 4D–O**). Considering the importance of the CD8+ T-cell infiltration level for neoantigen-targeted therapy and the prognosis of patients with ACGEJ, we conducted WGCNA to obtain CD8+ T-cell-related Hub genes. In addition, to gain a more comprehensive understanding of CD8+ T-cell infiltration, we assembled a relatively large-scale cohort of 93 Chinese ACGEJ patients with RNA-seq data and complete follow-up information by integrating our previously published data. In this dataset, clinical information included age, gender, TNM stage and the absolute abundance of CD8+ T cells (quantified by MCP-counter method) as well as survival status and OS. A soft-thresholding power of β = 4 was chosen to construct a scale-free network (scale-free *r* ^2^ = 0.88, slope = -1.62) (**Supplementary Figures 5A, D**). A total of 12 gene coexpression modules were identified from the hierarchical clustering tree (**Figures 5A, B**). As shown in the module–trait relationship heatmap (**Figure 5B**), the correlation coefficients and p values between module eigengenes and clinical traits were determined to identify which modules were associated with clinical information. The turquoise module was identified to be significantly positively related to the infiltration of CD8+ T cells in the ACGEJ samples (*r* = 0.61, *P* = 8x10^-11^). Furthermore, we applied GO and KEGG analyses to explore the biological functions of genes in the turquoise module. The GO analysis showed that the turquoise module genes were mainly related to the terms cytokine receptor activity, external side of the plasma membrane and T-cell activation (**Figure 5C**). The KEGG pathway analysis revealed that the genes were mainly enriched in the terms haematopoietic cell lineage, Th1/Th2 cell differentiation, Th17-cell differentiation, chemokine signalling pathway and cytokine–cytokine receptor interaction (**Figure 5D**). These results suggest that the turquoise module genes may be involved in the response to immunotherapy. To further investigate new potential targets for immunotherapies (immunosuppressive therapies or T-cell proliferation-inducing therapies), we used cut-off thresholds (GS > 0.6 and MM > 0.8; **Figure 5E**) to identify key genes in the turquoise module. We identified the ten most important Hub genes in the module that were related to CD8+ T-cell infiltration: *CCL5*, *CD2*, *CST7*, *GVINP1*, *GZMK*, *IL2RB*, *IKZF3*, *PLA2G2D*, *P2RY10* and *ZAP70* (**Figure 5F**).

### Construction and validation of the risk prediction model

**Figure 6:**
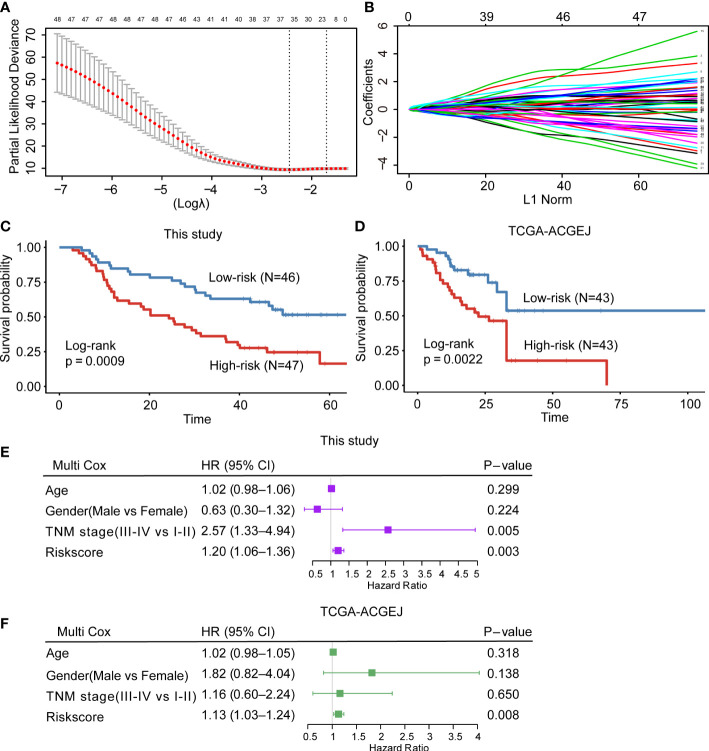
Construction and validation of the CD8+ T-cell infiltration-related prognostic prediction model. **(A, B)** LASSO Cox regression with 10-fold cross-validation to determine the prognostic value of 48 prognosis-related genes in the turquoise module. **(C, D)** Kaplan–Meier curve for the training cohort **(C)** and external validation cohort **(D)**. **(E, F)** Multivariate Cox regression analysis of clinicopathological factors and the risk score for our ACGEJ patients **(E)** and TCGA-ACGEJ patients **(F)**.

Previous studies have reported that high neoantigen load was associated with good survival in many cancers (13–16). To determine whether the potential neoantigens could predict OS in patients with ACGEJ, we divided patients into high and low groups based on the median of neoantigen load and the ratio of neoantigen to mutation. Regrettably, we found that neither neoantigen load nor the ratio of neoantigen to mutation was associated with patient OS (**Supplementary Figures 3E, F**). However, both methods, MCP-counter and xCell (**Supplementary Figure 3G** and **Figure 4N**), showed that the abundance of CD8+ T cells was positively correlated with the ratio of neoantigen to mutation (MCP-counter: r =0.28, P = 0.044; xCell: r =0.36, P =0.007). To further explore the prognostic value of the CD8+ T-cell infiltration-related module genes and identify potential prognostic factors of ACGEJ, we determined the prognostic value of the turquoise module genes. We obtained 48 genes significantly related to ACGEJ OS through univariate Cox regression analysis. After LASSO Cox and multivariate Cox regression analyses (**Figures 6A, B** and **Supplementary Figure 6A**), we identified seven genes (*ADAM28*, *ASPH*, *CAMK2N1*, *F2R*, *STAP1*, *TP53INP2*, *ZC3H3*) and used these genes to construct a prognosis prediction model with a training cohort containing our ACGEJ samples (n=93) and an external validation cohort containing TCGA ACGEJ samples (n=86). The risk score for all ACGEJ samples was calculated by the following formula: risk score = expression level of ZC3H3*1.83 + expression level of STAP1*1.25 + expression level of ADAM28*0.77+ expression level of TP53INP2*(-1.03) + expression level of CAMK2N1*1.05 + expression level of *F2R* *0.84 + expression level of *ASPH* *1.10. Taking the median risk score as the cut-off value, ACGEJ samples were divided into high- and low-risk score groups. The results showed that patients in the high-risk group had a significantly worse prognosis than those in the low-risk group in both the training cohort (*P*< 0.001, **Figure 6C**) and validation cohort (*P*< 0.01, **Figure 6D**). As shown in **Supplementary Figures 6B, C**, the area under the curve (AUC) was 0.729 in the training cohort and 0.712 in the validation cohort, which indicated that the risk model had good prediction performance. Next, multivariate Cox regression analysis confirmed that the risk score was an independent prognostic factor after adjusting for age, gender and TNM stage in both our ACGEJ cohort (HR =1.20, 95% CI = 1.06–1.36, *P*< 0.01; **Figure 6E**) and the TCGA-ACGEJ cohort (HR =1.13, 95% CI = 1.03–1.24, *P*< 0.01; **Figure 6F**). In summary, our results showed that the risk score can serve as an effective predictor for the risk classification of ACGEJ patients.

## Discussion

In this study, we determined the genomic alteration and neoantigen landscapes in samples collected from Chinese patients with ACGEJ. Comparing the genomic alterations in our 55 WES samples with those in 124 whole-genome sequencing samples revealed similar mutational signature patterns and chromosome arm level CNVs and showed that *TP53* was the only significant driver gene, *CCNE1* and *VEGFA* amplifications occurred frequently and were correlated with gene expression upregulation. However, our WES data seem to have fewer putative CNV driver genes, probably because of the smaller sample size. We found some differences between Siewert II and III samples, including differences in the mutational signatures SBS17a and SBS17b, copy number of *TNFRSF14*, the prevalence of the complex clonal pattern and neoantigen load. We identified of 58 shared tumor neoantigens, which could provide candidate targets for neoantigen-based targeted therapy. In addition, we identified 10 CD8+ T-cell infiltration-related Hub genes that may serve as immunotherapeutic biomarkers. Moreover, we constructed a risk prediction model based on CD8+ T-cell infiltration-related genes. Our findings in the present study provide a deeper understanding of ACGEJ that may lead to improved candidate target selection, and we proposed a novel prognostic prediction model.

We identified 11 recurrently mutated genes in two cohorts, 3 of which (*RNF43*, *MAP2K7* and *RHOA*) were newly discovered driver genes in ACGEJ. The E3 ubiquitin ligase RING finger protein 43 (RNF43) inhibits WNT signalling by ubiquitinating Frizzled receptors and targeting them for degradation (60). Loss of RNF43 function weakens the DNA damage response, leading to resistance to radiotherapy and chemotherapy in gastric cancer (61). *RNF43* was found to be frequently mutated in tumors with microsatellite instability and was identified as a significantly mutated driver gene in gastric adenocarcinoma (62). In our cohort, the vast majority of samples were microsatellite stable, and only 2% of samples had *RNF43* mutation. Mitogen-activated protein kinase 7 (*MAP2K7*) is an important tumor suppressor in gastric cancer, and frequent loss-of-function mutations activate the JNK pathway (63). Ras homologue family member A (*RHOA*) belongs to the Rho family of GTPases and is a driver gene in diffuse-type gastric carcinoma (64, 65). *RHOA* mutation was not found in the Chinese patients in this study, and its mutation might be related to differences in genetic or environmental risk factors, but this conclusion needs to be further verified in larger cohorts.

SBS17a and SBS17b have been reported to appear at an early stage of esophageal and gastric adenocarcinoma and to be related to gastric-oesophageal reflux (66, 67). SBS17a and SBS17b may be markers of oxidative damage and risk factors for chromosomal instability in ACGEJ (8, 68). SBS17b accounted for more somatic mutations than SBS17a in our cohort, which is in line with the phenomenon that 5′-C [T > G] T-3′ was the most common somatic single base substitution in ACGEJ samples. Due to differences in anatomical position, we believe that Siewert type III tumors are more likely to feature oxidative stress, which was supported by the result that the SBS17a and SBS17b signatures were significantly more prominent in Siewert type III samples than in Siewert type II samples. With high-depth (mean coverage 380X) WES, we confirmed that *CCNE1* and *VEGFA* are robust CNV driver genes in ACGEJ. We also identified *TP53* mutations and CNVs related to the p53/cell cycle and PI3K/AKT signalling pathways. By analysing clonal and subclonal profiles, we demonstrated that the complex clonal pattern was most common in ACGEJ samples, reflecting the intratumor heterogeneity in this cancer. A limitation of our study is that we could not show clonal evolution by drawing fishplots and Baysian trees in ACGEJ because of the difficulty in collecting matched pre- and post-treatment samples, paired primary and metastatic samples, or samples taken from multiple sites in an individual.

We also performed a comprehensive analysis of genomic alterations and transcriptomic changes to predict neoantigens. We found that the number of neoantigens produced by *SYNE1* mutation was second to that produced by *TP53* mutation. Although *SYNE1* was not identified as a driver gene in our ACGEJ samples, it has been identified as a significantly mutated gene in Western patients with ACGEJ and Chinese patients with gastric cancer (53, 69). As such, we plan to study the role of *SYNE1* mutation-derived neoantigens in future research. Based on analysis with two neoantigen-related databases, TSNAdb and IEDB, we obtained 58 neoantigens involving 50 genes (**Supplementary Table 10**) that may serve as candidate targets for neoantigen vaccines.

Several clinical trials have demonstrated that neoantigen vaccines based on dendritic cells, peptides and mRNAs can induce CD8+ T-cell-specific responses, emphasizing the considerable potential of neoantigens in immunotherapy (21, 22, 70). However, neoantigen vaccines spontaneously upregulate the expression of surface molecules (such as PD-1, TIM3 and CTLA-4) in neoantigen-specific T cells and/or PD-L1 in tumor cells, which in turn impedes the function of the neoantigen vaccines (71). Thus, a combination of neoantigen vaccines and immunosuppressive therapies to improve vaccine-induced T-cell responses is recommended. In addition, the combination of neoantigen vaccines and T-cell proliferation-inducing therapy, such as VB10.NEO plus NKTR-214, was confirmed to induce strong immunogenic CD8+ T-cell responses in preclinical models (27). Subsequently, based on WGCNA, we identified important Hub genes in ACGEJ positively associated with the infiltration of CD8+ T cells, providing new insights for research on the mechanisms of action of immunotherapy and offering potential targets for combination therapies in ACGEJ. We also constructed a risk prediction model based on the CD8+ T-cell infiltration -related genes, and the risk score showed good performance in predicting the OS of ACGEJ patients with in both the training cohort and the external validation cohort.

## Conclusion

Our study reveals the genomic characteristics and neoantigen features of ACGEJ. Through WGCNA, we established a CD8+ T-cell infiltration-related prediction model. Furthermore, many newly identified potential therapeutic targets can be pursued.

## Data availability statement

The original contributions presented in the study are included in the article/**Supplementary Materials**. Further inquiries can be directed to the corresponding author.

## Ethics statement

The studies involving human participants were reviewed and approved by the Institutional Review Board of Cancer Hospital, Chinese Academy of Medical Sciences, and Peking Union Medical College. The patients/participants provided their written informed consent to participate in this study.

## Author contributions

Y.Lao performed bioinformatics analysis. Y.W., J.Y., T.L., Y.M.,Y.Luo, Y.S., K.L. and X.Z. contributed to statistical analysis. X.N., Y.X. and C.Z. responded for sample collection and performed experiments. All authors contributed to the article and approved the submitted version.

## Funding

This project was supported by National Key Research and Development Program of China (2016YFC1302700 to CW), National Science Fund for Distinguished Young Scholars (81725015 to CW), Medical and Health Technology Innovation Project of Chinese Academy of Medical Sciences (2016-I2M-4-002 to CW, 2019-I2M-2-001 to DL and CW), Beijing Outstanding Young Scientist Program (BJJWZYJH01201910023027 to CW).

## Conflict of interest

The authors declare that the research was conducted in the absence of any commercial or financial relationships that could be construed as a potential conflict of interest.

The reviewer WL declared a shared affiliation with the authors to the handling editor at the time of review.

## Publisher’s note

All claims expressed in this article are solely those of the authors and do not necessarily represent those of their affiliated organizations, or those of the publisher, the editors and the reviewers. Any product that may be evaluated in this article, or claim that may be made by its manufacturer, is not guaranteed or endorsed by the publisher.

## Supplementary material

Supplementary Figure 1 The landscape of somatic mutations and mutational signatures of ACGEJ. **(A)** Violin plots comparing tumor mutation burden differences across the two cohorts. **(B, C)** Box plots comparing tumor mutation burden in our ACGEJ samples with different Siewert types **(B)** or differentiation grades **(C)**. **(D)** Comparison of distribution of non-synonymous TP53 somatic mutations between our tumor samples and TCGA/Tumor Portal samples. **(E)** Mutational spectra of our ACGEJ samples. *P* values were derived from Wilcoxon rank-sum tests; *****P<* 0.0001; ns, not significant; G1: well differentiated; G2: moderately differentiated; G3: poorly differentiated or undifferentiated.

Supplementary Figure 2
**(A, B)** Box plots comparing gene level CNVs in our ACGEJ samples with different Siewert types **(A)** or differentiation grades **(B)**. *P* values were derived from Wilcoxon rank-sum tests. Somatic CNVs are limited to homozygous deletion and high-level amplifications (GISTIC score of -2 and 2 respectively).

Supplementary Figure 3
**(A, C)** Box plots comparing number of neoantigens in our ACGEJ samples with different TNM stages **(A)** and differentiation grades **(C)**. (B, D) Box plots comparing number of high-affinity neoantigens in ACGEJ samples with different TNM stages **(B)** and differentiation grades **(D)**. NeoAgs: neoantigens. *P* values were derived from Wilcoxon rank-sum tests. **(E, F)** Kaplan–Meier curve for neoantigen load **(E)** and the ratio of neoantigen to mutation **(F)**. **(G)** Correlations between CD8+ T cell infiltration level (quantified by MCP-counter method) and the ratio of neoantigen to mutation (Spearman’s correlation test).

Supplementary Figure 4
**(A–O)** Association of the ratio of neoantigen to mutation and immune score, stromal score and microenvironment score and TME cells (quantified by xCell method) (Spearman’s correlation test).

Supplementary Figure 5
**(A)** Network topology was plotted by different soft thresholding powers (x-axis) and correlation coefficient between log (k) and log [P(k)] (y-axis). The red line represents a correlation coefficient of 0.9. An approximate soft-thresholding powers, β=4, was obtained. **(B)** Mean connectivity under different soft-thresholding powers. **(C, D)** Assessing the scale-free topology when β was set to 4.

Supplementary Figure 6
**(A)** Multivariate Cox regression analysis of 17 OS-related factors. **(B, C)** ROC curve of the training cohort **(B)** and external validation cohort **(C)**.
