# Supplementary material for: Characterization of genomic alterations and neoantigens and analysis of immune infiltration identified therapeutic and prognostic biomarkers in adenocarcinoma at the gastroesophageal junction
Source: Front Oncol. 2022 Nov 11;12:941868. doi: 10.3389/fonc.2022.941868 (PMC9691957; doi:10.3389/fonc.2022.941868)
Supplement: Supplementary Figure 1 — The landscape of somatic mutations and mutational signatures of ACGEJ. (A) Violin plots comparing tumor mutation burden differences across the two cohorts. (B, C) Box plots comparing tumor mutation burden in our ACGEJ samples with different Siewert types (B) or differentiation grades (C). (D) Comparison of distribution of non-synonymous TP53 somatic mutations between our tumor samples and TCGA/Tumor Portal samples. (E) Mutational spectra of our ACGEJ samples. P values were derived from Wilcoxon rank-sum tests; ****P< 0.0001; ns, not significant; G1: well differentiated; G2: moderately differentiated; G3: poorly differentiated or undifferentiated. [file DataSheet_1.zip › Suppl Figure legends.docx]

**Supplementary Figure Legends**

**Figure S1** The landscape of somatic mutations and mutational signatures of ACGEJ.

(**A**) Violin plots comparing tumor mutation burden differences across the two cohorts. (**B-C**) Box plots comparing tumor mutation burden in our ACGEJ samples with different Siewert types(**B**) or differentiation grades(**C**). (**D**) Comparison of distribution of non-synonymous TP53 somatic mutations between our tumor samples and TCGA/Tumor Portal samples. (**E**) Mutational spectra of our ACGEJ samples. *P* values were derived from Wilcoxon rank-sum tests; *****P* < 0.0001; ns, not significant; G1: well differentiated; G2: moderately differentiated; G3: poorly differentiated or undifferentiated.

**Figure S2**

(**A-B**) Box plots comparing gene level CNVs in our ACGEJ samples with different Siewert types(**A**) or differentiation grades(**B**). *P* values were derived from Wilcoxon rank-sum tests. Somatic CNVs are limited to homozygous deletion and high-level amplifications (GISTIC score of -2 and 2 respectively).

**Figure S3**

(**A, C**) Box plots comparing number of neoantigens in our ACGEJ samples with different TNM stages (**A**) and differentiation grades (**C**). **(B, D**) Box plots comparing number of high-affinity neoantigens in ACGEJ samples with different TNM stages (**B**) and differentiation grades (**D**). NeoAgs: neoantigens. *P* values were derived from Wilcoxon rank-sum tests. (**E-F**) Kaplan–Meier curve for neoantigen load (**E**) and the ratio of neoantigen to mutation (**F**). (**G**) Correlations between CD8+ T cell infiltration level (quantified by MCP-counter method) and the ratio of neoantigen to mutation (Spearman’s correlation test).

**Figure S4**

**(A-O)** Association of the ratio of neoantigen to mutation and immune score, stromal score and microenvironment score and TME cells (quantified by xCell method) (Spearman’s correlation test).

**Figure S5**

(**A**) Network topology was plotted by different soft thresholding powers (x-axis) and correlation coefficient between log (k) and log [P(k)] (y-axis). The red line represents a correlation coefficient of 0.9. An approximate soft-thresholding powers, β=4, was obtained. (**B**) Mean connectivity under different soft-thresholding powers. (**C-D**) Assessing the scale-free topology when β was set to 4.

**Figure S6**

(**A**) Multivariate Cox regression analysis of 17 OS-related factors.

(**B-C**) ROC curve of the training cohort (**B**) and external validation cohort (**C**).
