# Supplementary figures and images for: Characterization of genomic alterations and neoantigens and analysis of immune infiltration identified therapeutic and prognostic biomarkers in adenocarcinoma at the gastroesophageal junction
Source: Front Oncol. 2022 Nov 11;12:941868. doi: 10.3389/fonc.2022.941868 (PMC9691957; doi:10.3389/fonc.2022.941868)

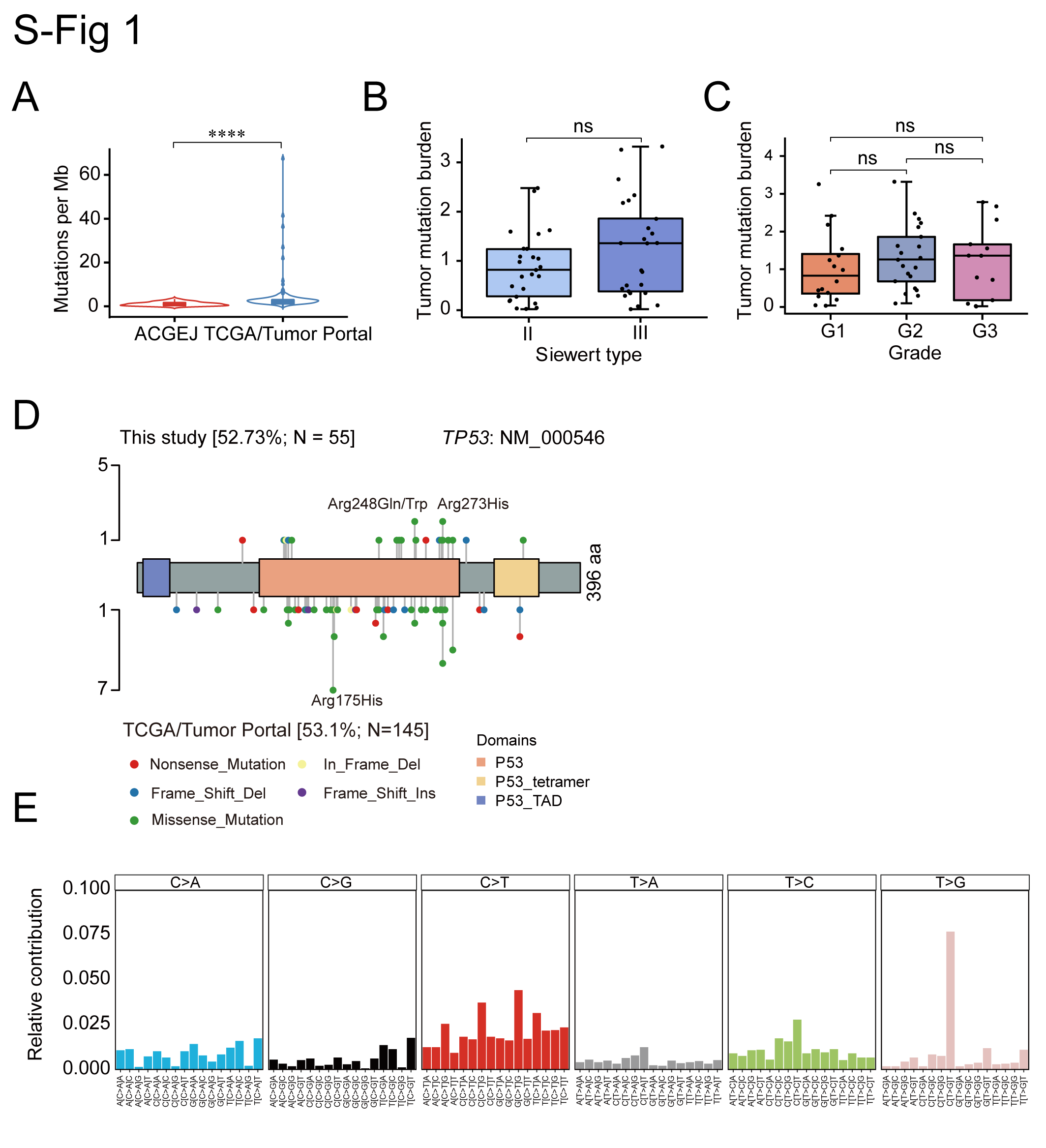

Supplement: Supplementary Figure 1 — The landscape of somatic mutations and mutational signatures of ACGEJ. (A) Violin plots comparing tumor mutation burden differences across the two cohorts. (B, C) Box plots comparing tumor mutation burden in our ACGEJ samples with different Siewert types (B) or differentiation grades (C). (D) Comparison of distribution of non-synonymous TP53 somatic mutations between our tumor samples and TCGA/Tumor Portal samples. (E) Mutational spectra of our ACGEJ samples. P values were derived from Wilcoxon rank-sum tests; ****P< 0.0001; ns, not significant; G1: well differentiated; G2: moderately differentiated; G3: poorly differentiated or undifferentiated. [file DataSheet_1.zip › Lao et al_Suppl Fig 1.tif]

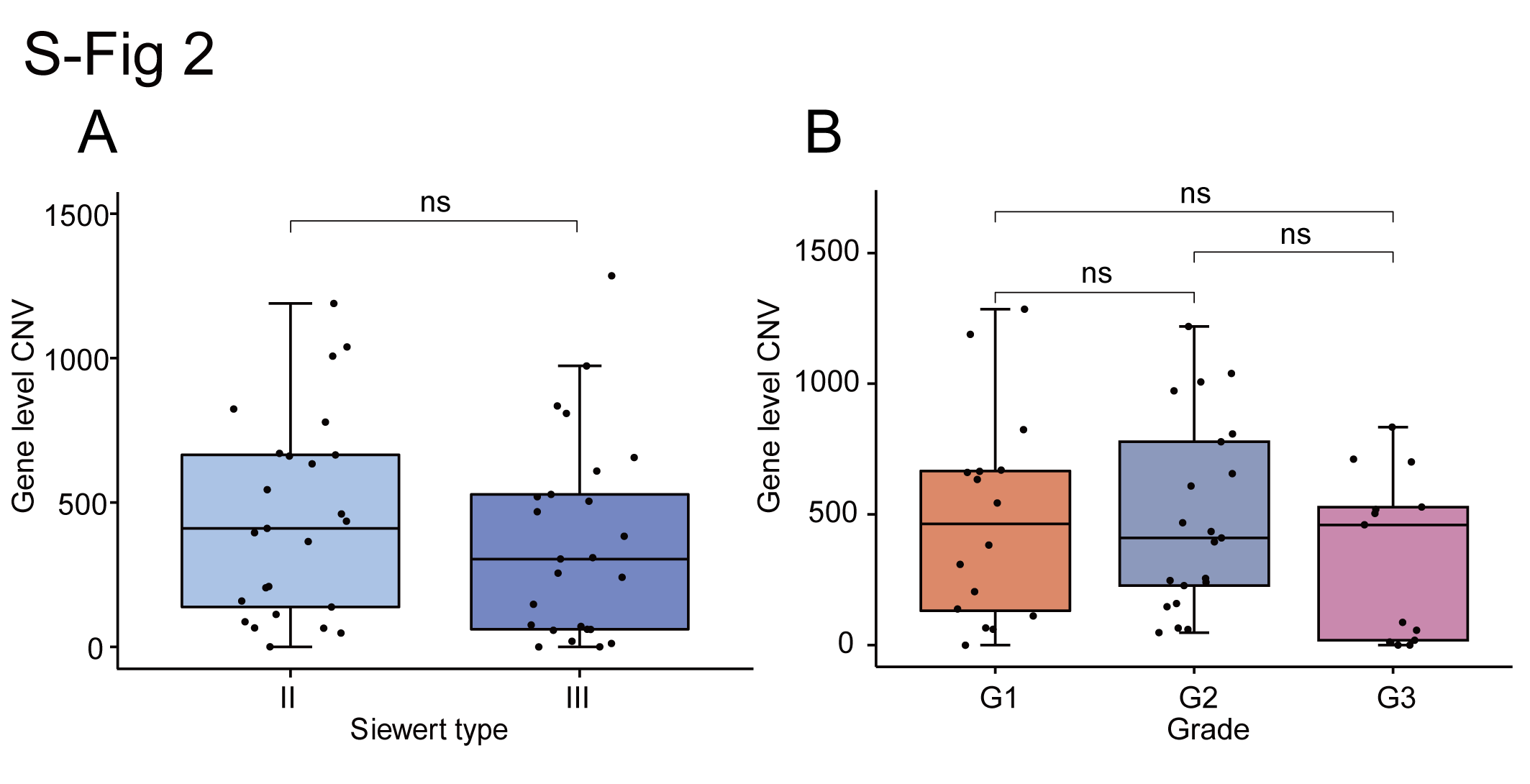

Supplement: Supplementary Figure 1 — The landscape of somatic mutations and mutational signatures of ACGEJ. (A) Violin plots comparing tumor mutation burden differences across the two cohorts. (B, C) Box plots comparing tumor mutation burden in our ACGEJ samples with different Siewert types (B) or differentiation grades (C). (D) Comparison of distribution of non-synonymous TP53 somatic mutations between our tumor samples and TCGA/Tumor Portal samples. (E) Mutational spectra of our ACGEJ samples. P values were derived from Wilcoxon rank-sum tests; ****P< 0.0001; ns, not significant; G1: well differentiated; G2: moderately differentiated; G3: poorly differentiated or undifferentiated. [file DataSheet_1.zip › Lao et al_Suppl Fig 2.tif]

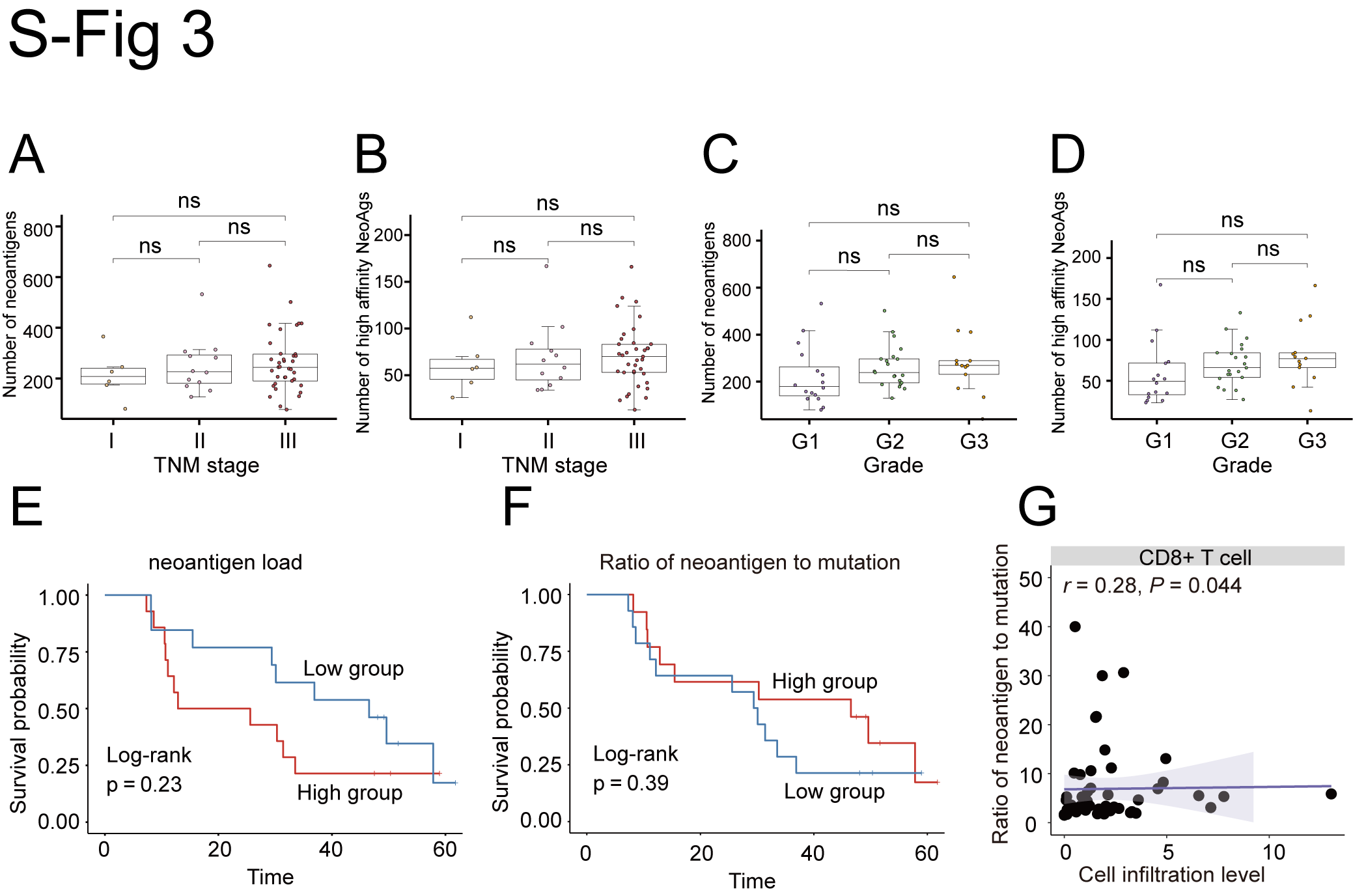

Supplement: Supplementary Figure 1 — The landscape of somatic mutations and mutational signatures of ACGEJ. (A) Violin plots comparing tumor mutation burden differences across the two cohorts. (B, C) Box plots comparing tumor mutation burden in our ACGEJ samples with different Siewert types (B) or differentiation grades (C). (D) Comparison of distribution of non-synonymous TP53 somatic mutations between our tumor samples and TCGA/Tumor Portal samples. (E) Mutational spectra of our ACGEJ samples. P values were derived from Wilcoxon rank-sum tests; ****P< 0.0001; ns, not significant; G1: well differentiated; G2: moderately differentiated; G3: poorly differentiated or undifferentiated. [file DataSheet_1.zip › Lao et al_Suppl Fig 3.tif]

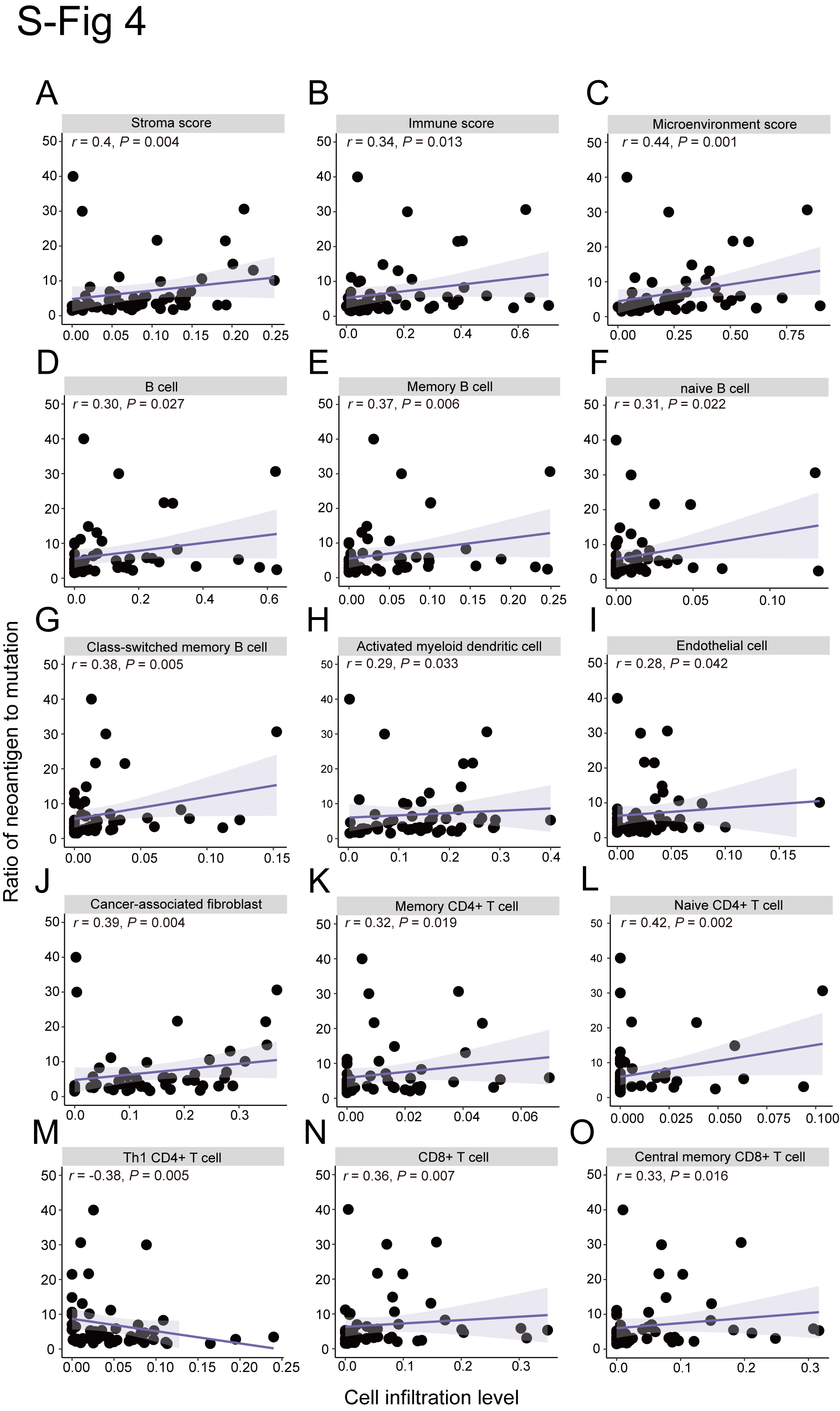

Supplement: Supplementary Figure 1 — The landscape of somatic mutations and mutational signatures of ACGEJ. (A) Violin plots comparing tumor mutation burden differences across the two cohorts. (B, C) Box plots comparing tumor mutation burden in our ACGEJ samples with different Siewert types (B) or differentiation grades (C). (D) Comparison of distribution of non-synonymous TP53 somatic mutations between our tumor samples and TCGA/Tumor Portal samples. (E) Mutational spectra of our ACGEJ samples. P values were derived from Wilcoxon rank-sum tests; ****P< 0.0001; ns, not significant; G1: well differentiated; G2: moderately differentiated; G3: poorly differentiated or undifferentiated. [file DataSheet_1.zip › Lao et al_Suppl Fig 4.tif]

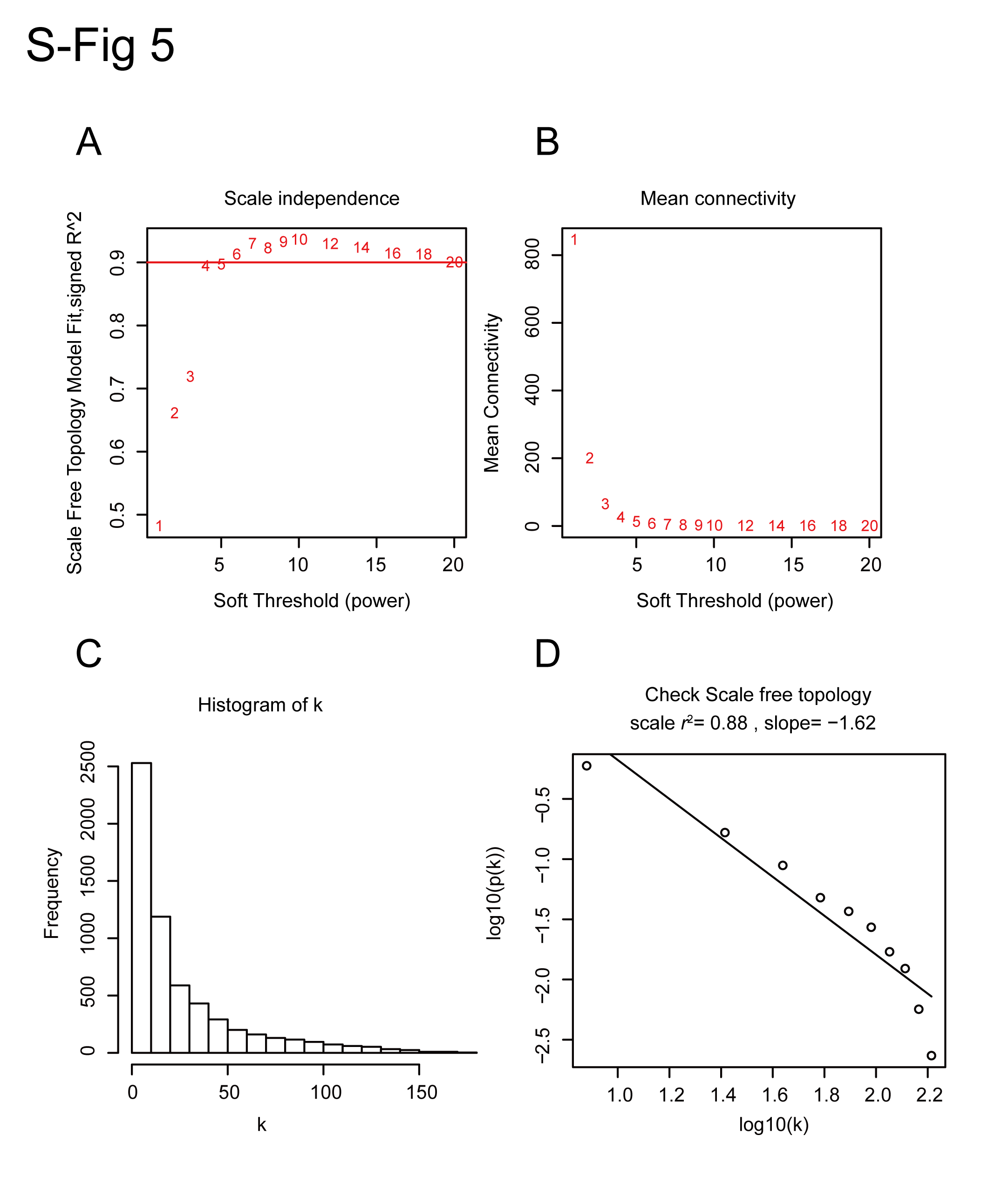

Supplement: Supplementary Figure 1 — The landscape of somatic mutations and mutational signatures of ACGEJ. (A) Violin plots comparing tumor mutation burden differences across the two cohorts. (B, C) Box plots comparing tumor mutation burden in our ACGEJ samples with different Siewert types (B) or differentiation grades (C). (D) Comparison of distribution of non-synonymous TP53 somatic mutations between our tumor samples and TCGA/Tumor Portal samples. (E) Mutational spectra of our ACGEJ samples. P values were derived from Wilcoxon rank-sum tests; ****P< 0.0001; ns, not significant; G1: well differentiated; G2: moderately differentiated; G3: poorly differentiated or undifferentiated. [file DataSheet_1.zip › Lao et al_Suppl Fig 5.tif]

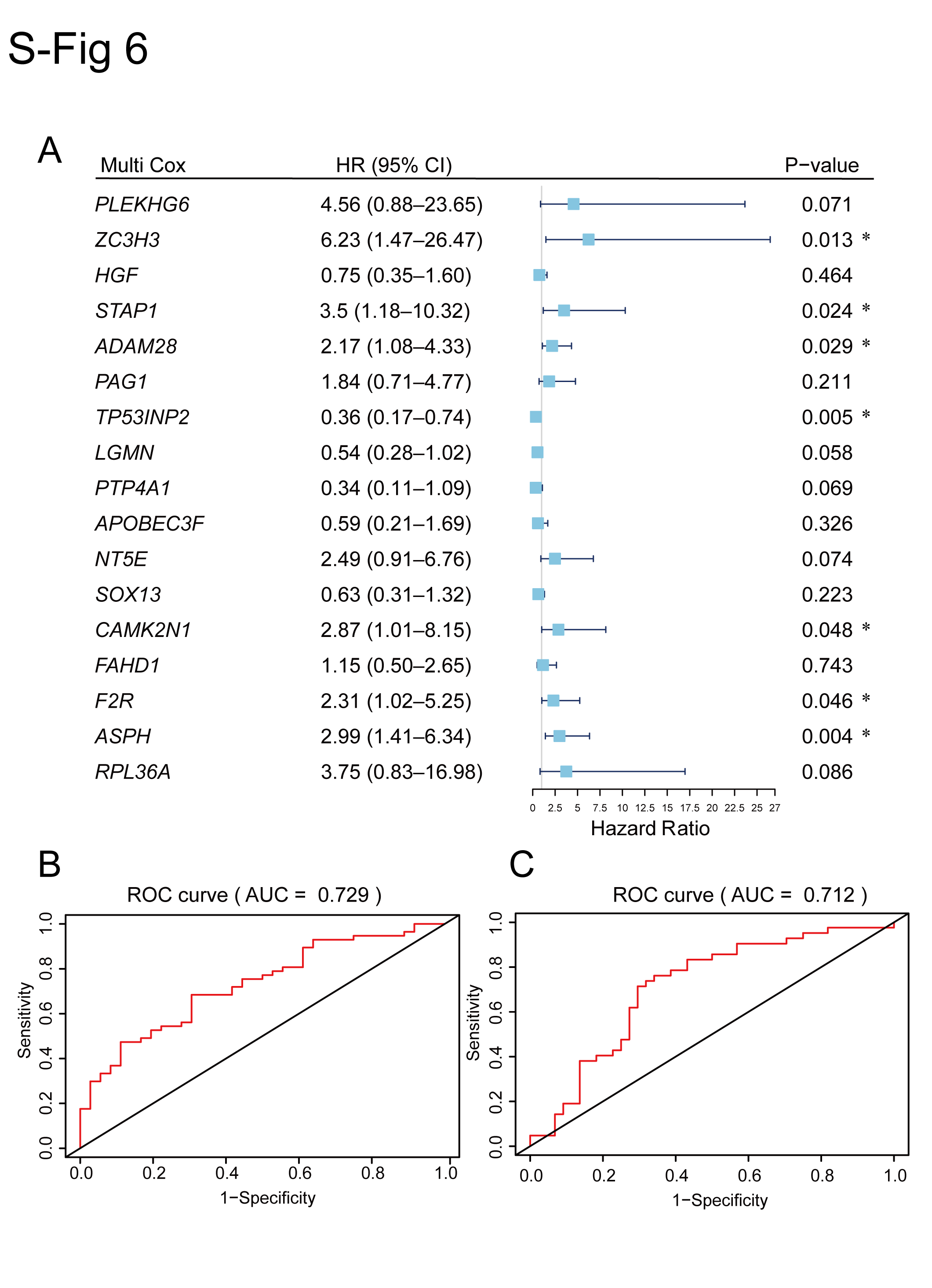

Supplement: Supplementary Figure 1 — The landscape of somatic mutations and mutational signatures of ACGEJ. (A) Violin plots comparing tumor mutation burden differences across the two cohorts. (B, C) Box plots comparing tumor mutation burden in our ACGEJ samples with different Siewert types (B) or differentiation grades (C). (D) Comparison of distribution of non-synonymous TP53 somatic mutations between our tumor samples and TCGA/Tumor Portal samples. (E) Mutational spectra of our ACGEJ samples. P values were derived from Wilcoxon rank-sum tests; ****P< 0.0001; ns, not significant; G1: well differentiated; G2: moderately differentiated; G3: poorly differentiated or undifferentiated. [file DataSheet_1.zip › Lao et al_Suppl Fig 6.tif]
